# Artificial Intelligence in Implant Dentistry: Clinical Validity, Diagnostic Performance, Surgical Planning, and Medico-Legal Implications—A Narrative Review

**DOI:** 10.3390/dj14070389

**Published:** 2026-06-23

**Authors:** Alfonso Acerra, Angelo Aliberti, Alessandra Amato, Anna Eccellente, Alessandro Santurro, Francesco Giordano

**Affiliations:** 1 Department of Medicine, Surgery and Dentistry, University of Salerno, 84084 Salerno, Italy; aaleamato@gmail.com (A.A.); asanturro@unisa.it (A.S.); frgiordano@unisa.it (F.G.); 2 Department of Neurosciences, Reproductive Sciences and Odontostomatological Sciences, University of Naples Federico II, 80131 Naples, Italy; anna.eccellente@aoufedericoii.unina.it

**Keywords:** artificial intelligence, implant dentistry, dental implants, cone-beam computed tomography, implant planning, surgical navigation, predictive modeling, medico-legal implications

## Abstract

**Background**: Artificial intelligence (AI) is increasingly being integrated into implant dentistry, where clinical decision-making depends on the interpretation of complex radiographic and patient-specific data. Although multiple applications have been proposed across diagnostic imaging, treatment planning, intraoperative support and outcome prediction, their clinical validity and real-world applicability remain incompletely defined and their use raises relevant medico-legal considerations. **Methods**: A narrative review was conducted through a structured search of PubMed/MEDLINE, Scopus, and Web of Science, including English-language studies published between 2010 and February 2026. Clinical and experimental studies, as well as relevant reviews addressing AI applications in implant dentistry, were included. A qualitative thematic synthesis was performed due to methodological heterogeneity. **Results**: AI applications are mainly concentrated in diagnostic imaging, particularly CBCT analysis, where high levels of performance are consistently reported. In treatment planning, systems support specific decision-making tasks rather than comprehensive strategies, while intraoperative applications are integrated into navigation and robotic systems to improve procedural accuracy. Predictive models for implant outcomes have been developed, although their reliability remains influenced by dataset variability and study design. **Conclusions**: AI currently represents a supportive tool in implant dentistry, with greater applicability in standardized tasks. Its integration into complex clinical decision-making remains limited, highlighting the need for clinically oriented validation and cautious implementation in practice.

## 1. Introduction

In recent years, artificial intelligence (AI) has progressively emerged as a transformative technology in healthcare, driven by its ability to process large-scale datasets, identify complex and non-linear patterns, and support clinical decision-making processes [1]. Machine learning and deep learning algorithms are increasingly integrated into diagnostic and therapeutic workflows, contributing to improvements in diagnostic accuracy, clinical efficiency, and the development of personalized treatment strategies [2]. Within this evolving landscape, AI has demonstrated broad applicability across multiple medical disciplines, particularly in imaging-based fields, where data-driven approaches can enhance the interpretation of complex clinical information [3].

Among dental specialties, implant dentistry represents one of the most promising areas for the application of AI. Implant therapy is inherently complex and requires the integration of multiple variables, including anatomical conditions, prosthetic requirements, surgical considerations, and patient-related factors [4,5]. Clinical decision-making in implantology is largely dependent on the accurate interpretation of radiographic imaging, especially cone-beam computed tomography (CBCT), as well as on the identification of critical anatomical structures and the evaluation of bone quantity and quality [6,7]. This combination of high data density and decision complexity makes implant dentistry particularly suitable for the implementation of AI-based systems.

A growing body of evidence indicates that AI can achieve high levels of diagnostic performance in implant dentistry. AI-based algorithms have been successfully applied to the automated detection and segmentation of anatomical structures, such as the mandibular canal, maxillary sinus, and peri-implant bone, as well as to the identification and classification of dental implants in radiographic images [8]. In several of these applications, AI systems have demonstrated accuracy comparable to, and in some cases exceeding, that of experienced clinicians, with reported performance metrics frequently exceeding 90% [9]. Beyond diagnostic applications, AI is increasingly being explored as a decision-support system in implant planning [10]. Advanced predictive models can integrate clinical, radiological, and patient-related data to assist in implant positioning, prosthetically driven treatment planning, and risk assessment. In this context, AI has shown potential in predicting implant success, estimating the likelihood of complications, and supporting more individualized therapeutic strategies [11]. Furthermore, the integration of AI within digital workflows, including three-dimensional imaging, computer-guided surgery, and dynamic navigation systems, is contributing to the development of more precise and reproducible implant procedures [12,13].

The role of AI is also expanding into the intraoperative phase, where emerging technologies combining real-time navigation, augmented visualization, and robotic assistance aim to enhance surgical accuracy and reduce operator-dependent variability [14]. These systems have the potential to improve the predictability of implant placement and to minimize intraoperative risks [4]. However, despite these promising developments, the clinical translation of AI in implant dentistry remains far from fully established. Indeed, a substantial portion of the available literature is characterized by methodological heterogeneity, limited external validation, and reliance on retrospective or highly selected datasets. Moreover, most studies predominantly report technical performance metrics, such as accuracy, sensitivity, or specificity, without adequately addressing their clinical interpretability or real-world applicability [15]. Consequently, high algorithmic performance does not necessarily translate into clinically meaningful or generalizable outcomes, raising important concerns regarding the actual validity and effectiveness of AI-based systems in implantology [10].

In parallel with these technical and clinical considerations, the integration of AI into implant dentistry introduces significant ethical, legal, and medico-legal challenges that are inherently linked to its clinical use [16,17]. The adoption of algorithm-assisted decision-making systems raises fundamental questions regarding professional responsibility, particularly in cases of diagnostic or therapeutic errors, where the attribution of liability between clinician and technology remains unclear [18]. Issues related to algorithmic transparency, often referred to as the “black box” problem, further limit the clinician’s ability to critically evaluate and justify AI-generated outputs within both clinical and legal frameworks [19]. In addition, the use of large datasets for model training requires strict attention to patient data protection, cybersecurity, and regulatory compliance [20]. The process of informed consent also becomes more complex in the context of AI-assisted care, as patients must be adequately informed about the role, benefits, and limitations of these technologies in clinical decision-making [21].

Despite the rapid growth of research in this field, the literature currently lacks a comprehensive and critically oriented synthesis specifically addressing the clinical validity, interpretative limits, and medico-legal implications of AI in implant dentistry. Most available reviews provide descriptive overviews of technological advancements without offering a structured evaluation of their translational relevance and integration into clinical practice [4,10,14]. Therefore, the aim of this narrative review is to critically analyze the current evidence on the application of artificial intelligence in implant dentistry, with particular emphasis on its role in diagnostic processes, surgical planning, and intraoperative support. In addition, this review aims to examine the medico-legal implications associated with the use of AI-based systems, focusing on issues of professional liability, data governance, algorithmic transparency, and informed consent. By adopting a clinically oriented and methodologically critical perspective, this work seeks to provide a structured framework for the interpretation and appropriate integration of AI in contemporary implant dentistry.

## 2. Materials and Methods

### 2.1. Literature Search Strategy

This narrative review was designed to provide a critical and clinically oriented synthesis of the current literature on the application of artificial intelligence (AI) in implant dentistry, with particular emphasis on its diagnostic performance, role in surgical planning, intraoperative support, and medico-legal implications. Given the rapid evolution of AI technologies and the heterogeneity of study designs, datasets, and reported outcomes, a narrative review approach was considered the most appropriate methodology to explore issues related to clinical validity, interpretative limits, and translational relevance, rather than to perform a quantitative synthesis.

A structured, non-systematic literature search was conducted using the PubMed/MEDLINE, Scopus, and Web of Science databases. The search included articles published from January 2010 to February 2026 and was restricted to English-language publications. This time frame was selected to encompass the period during which artificial intelligence, and, in particular, deep learning techniques, have been progressively integrated into dental research and clinical practice.

Search terms were combined using Boolean operators and included: “artificial intelligence”, “machine learning”, “deep learning”, “implant dentistry”, “dental implants”, “implant planning”, “guided implant surgery”, “CBCT”, “cone-beam computed tomography”, “image analysis”, “diagnostic accuracy”, “treatment planning”, “surgical navigation”, “robotic implantology”, “implant success prediction”, “risk assessment”, “peri-implantitis prediction”, “clinical decision support systems”, “medico-legal implications”, “legal responsibility”, “data protection”, and “informed consent”.

To ensure comprehensive coverage of the literature, a supplementary manual search was performed by screening the reference lists of all relevant articles identified in the electronic search. Additionally, targeted manual searches were conducted within key journals in the fields of implant dentistry, digital dentistry, and artificial intelligence in healthcare to identify high-impact and seminal studies that may not have been captured through database searches alone.

Given the narrative nature of this review, the search strategy was designed to ensure comprehensive coverage of relevant literature rather than strict reproducibility. Nevertheless, predefined eligibility criteria and thematic domains were established before study selection to enhance methodological consistency.

### 2.2. Eligibility Criteria and Study Selection

Eligible publications included original research articles, clinical studies, retrospective and prospective investigations, as well as narrative and systematic reviews addressing the application of artificial intelligence (AI) in implant dentistry. Experimental in vitro studies were included only when directly related to clinically relevant AI applications in implantology, such as radiographic image analysis, implant planning accuracy, surgical guidance, navigation systems, robotic-assisted procedures, and implant-related digital workflows.

Studies were considered eligible if they investigated AI-based systems applied to diagnostic processes, including radiographic interpretation and anatomical structure identification, implant treatment planning, intraoperative support technologies, or predictive models for treatment outcomes and complications. Studies focusing on two-dimensional and three-dimensional imaging modalities, including panoramic radiography and cone-beam computed tomography (CBCT), as well as investigations involving computer-guided surgery, dynamic navigation, and robotic implantology, were also included.

Narrative and systematic reviews were considered to contextualize technological developments, methodological approaches, and medico-legal considerations related to AI in implant dentistry.

Studies were excluded if they were not directly related to implant dentistry, if they focused exclusively on other dental specialties without clear applicability to implantology, or if they lacked sufficient methodological detail to allow meaningful interpretation of AI model performance and clinical relevance. Purely technical or engineering-oriented studies without direct clinical or translational relevance were not considered. In addition, experimental studies not specifically addressing implant-related diagnostic, planning, surgical, or outcome-oriented AI applications were excluded.

Study selection was independently performed by two authors (A.AC. and A.AL.). Titles, abstracts, and full-text articles were evaluated according to the predefined eligibility criteria and thematic domains established for this review. Any disagreements regarding study inclusion were resolved through discussion and consensus. Although this review followed a narrative design, these procedures were adopted to enhance consistency and minimize potential selection bias. Following the initial database search, titles and abstracts were screened for thematic relevance to AI applications in implant dentistry. After removal of duplicates, potentially eligible studies were assessed through full-text evaluation to determine their methodological contribution and alignment with the scope of this narrative review. The literature search identified a broad body of relevant literature across the selected databases. Approximately 65 studies were identified as particularly relevant for the qualitative synthesis and critical discussion of the predefined thematic domains. Given the narrative design of this review, study selection was not intended to follow a formal PRISMA-based process. Instead, studies were selected according to their relevance to the predefined thematic domains and their ability to contribute to the critical discussion of the clinical applicability, methodological limitations, and medico-legal implications of AI in implant dentistry. The main reasons for exclusion were lack of direct relevance to implant dentistry, absence of clear clinical or translational applicability, insufficient methodological detail, or a focus on purely technical aspects without meaningful connection to implant-related diagnosis, planning, surgical support, or outcome prediction.

In accordance with the narrative design of this study, no formal risk-of-bias assessment tools were applied. Instead, emphasis was placed on qualitative appraisal of the selected literature, focusing on key aspects such as dataset characteristics, model development and validation strategies, performance metrics, and the clinical interpretability of the reported outcomes.

### 2.3. Data Analysis and Synthesis

Given the marked heterogeneity in study design, AI methodologies, datasets, and reported outcome measures, a quantitative synthesis or meta-analysis was not considered methodologically appropriate. Instead, the selected literature was analyzed and synthesized using a thematic and clinically oriented approach.

Studies were grouped according to their primary field of application within implant dentistry, including diagnostic applications and radiographic analysis; implant planning and decision-support systems; intraoperative guidance and surgical assistance; predictive models for treatment outcomes and complications; and medico-legal and ethical implications of AI use in implantology. Within each thematic area, studies were evaluated qualitatively based on methodological characteristics, including dataset composition, model development and validation strategies, and reported performance metrics. Attention was given to the clinical interpretability of results and to the extent to which the reported outcomes could be considered applicable to real-world clinical scenarios.

### 2.4. Clinical Interpretation of AI Evidence

To complement the thematic synthesis, a qualitative approach was adopted to support a clinically oriented interpretation of the included studies. Given the narrative design of this review and the marked heterogeneity of study designs, datasets, and reported outcomes, this approach was not intended as a formal methodological assessment, but rather as a pragmatic framework to distinguish between technical performance and potential clinical applicability.

Three main aspects were considered. First, the type of validation was evaluated, differentiating studies based on internal validation, external validation, or, where available, prospective clinical assessment. Second, dataset characteristics were examined, including sample size, single-center or multicenter origin, variability in imaging conditions, and the representativeness of patient populations. Third, the nature of the reported outcomes was analyzed, distinguishing between technical performance metrics, such as accuracy, sensitivity, specificity, Dice similarity coefficient, or area under the receiver operating characteristic curve, and clinically meaningful outcomes, such as diagnostic reliability, implant positioning accuracy, reduction in surgical deviations, or prediction of complications.

Based on these elements, AI applications were qualitatively interpreted according to their level of clinical applicability. Most studies were considered either experimental, when based on proof-of-concept, in vitro, or simulation settings, or technically validated, when showing high performance under controlled conditions but with limited external or prospective validation. Only a limited number of studies approached potential clinical applicability, typically when supported by external validation or preliminary clinical evaluation.

## 3. Results

The analysis of the available literature highlights a rapidly expanding and heterogeneous application of artificial intelligence (AI) in implant dentistry. AI-based systems have been increasingly implemented across multiple phases of implant therapy, including diagnostic assessment, treatment planning, intraoperative guidance, and outcome prediction [22]. The identified studies reported a wide range of AI applications across diagnostic imaging, implant planning, intraoperative support, and predictive modeling. Considerable variability was observed in study design, methodological approaches, datasets, and validation strategies. Reported performance metrics should be interpreted with caution, as technical accuracy achieved under controlled experimental conditions does not necessarily translate into real-world clinical effectiveness. The distinction between algorithmic performance and clinical applicability remains a recurring theme across the available literature.

Across the included studies, AI applications can be broadly categorized into diagnostic imaging, implant planning, intraoperative support, predictive modeling, and medico-legal considerations. While high levels of technical performance are frequently reported, the extent to which these findings translate into consistent and generalizable clinical benefits remains a central issue.

### 3.1. AI in Diagnostic Imaging and Radiographic Analysis in Implant Dentistry

AI in diagnostic imaging represents one of the most frequently investigated areas within implant dentistry. The available literature is largely centered on the analysis of radiographic datasets, including cone-beam computed tomography (CBCT) and two-dimensional imaging modalities, in which AI-based systems have been developed for detection, segmentation, and classification tasks [23]. Across published studies, deep learning approaches, particularly convolutional neural networks, are the models most commonly employed for imaging-based applications. These systems have been used to identify and delineate anatomical structures relevant to implant therapy, including the mandibular canal, maxillary sinus, alveolar bone contours, and adjacent dental structures [24]. In this context, automated segmentation of anatomical regions is frequently reported, especially in CBCT-based workflows, where volumetric imaging data allow detailed structural representation [25].

A substantial portion of the literature describes segmentation-based approaches as part of radiographic analysis. These methods are typically evaluated in terms of agreement with expert annotations or segmentation accuracy metrics and are reported in both two-dimensional and three-dimensional imaging contexts, with a higher prevalence in CBCT-based studies [26]. Beyond anatomical analysis, imaging-based systems have also been applied to implant-specific diagnostic tasks, including the recognition and classification of dental implants from radiographic images [27]. Studies report that machine learning and deep learning models can differentiate implant systems or implant-related radiographic features when appropriately annotated datasets are available [28]. In addition, several investigations have explored the detection of peri-implant radiographic changes, such as marginal bone loss, peri-implant radiolucencies, and variations in bone density patterns, occurring in response to inflammatory conditions, peri-implant tissue breakdown, and biomechanical loading related to mandibular function [29,30].

Model performance is commonly reported using metrics such as accuracy, sensitivity, and specificity. Imaging-based studies frequently describe high performance, particularly for convolutional neural network architectures applied to standardized datasets [31]. However, results vary across studies, and direct comparison is limited by differences in imaging modality, annotation protocols, outcome definitions, and validation strategies [32].

Several studies also highlight the influence of dataset characteristics on model performance. Many investigations are based on retrospective datasets obtained from single institutions, often including relatively homogeneous imaging conditions and limited variability in patient populations [33]. Radiographic acquisition parameters, including voxel size, field of view, and reconstruction settings in CBCT imaging, are described as factors that may affect model behavior [34].

Another aspect reported across studies concerns the definition of reference standards. In most imaging-based investigations, ground truth is established through expert annotation, which may vary depending on operator experience and diagnostic criteria [35]. This variability is particularly relevant for subtle radiographic findings, such as early peri-implant changes, where interpretation may not be consistent across studies. Most studies in this domain were conducted using retrospective datasets, and validation procedures were performed under controlled research conditions.

### 3.2. AI in Implant Planning and Decision-Making

AI has been increasingly incorporated into implant planning and decision-support within contemporary digital workflows in implant dentistry [24]. The available literature describes a range of applications involving the analysis of preoperative imaging, support in anatomical and prosthetic assessment, and the integration of digital datasets within planning environments [36].

A substantial portion of studies focuses on preoperative planning supported by radiographic imaging, particularly CBCT. In these investigations, AI-based systems are used to assist in the evaluation of anatomical conditions relevant to implant placement, including bone morphology, available bone volume, and spatial relationships between implant sites and adjacent anatomical structures [37]. In several studies, outputs derived from image analysis, such as anatomical delineation or bone characterization, are incorporated into digital planning workflows, contributing to the definition of implant positioning and procedural parameters [38].

The literature also describes approaches aimed at supporting specific decision-making steps within the planning phase [12]. These include models developed to assist in the selection of drilling protocols, classification of surgical scenarios, and estimation of implant site characteristics based on radiographic features [39]. In these contexts, systems are generally designed to process structured datasets and generate outputs related to predefined planning parameters rather than to provide complete treatment plans. Previous narrative study similarly characterizes these approaches as tools intended to support planning-related decisions [10].

Another group of studies addresses the integration of these systems within broader digital workflows that combine multiple sources of patient data, including CBCT imaging, intraoral scans, and, in some cases, prosthetic datasets [40]. Within these environments, such technologies are described as contributing to data processing, alignment, or automation of specific planning steps. Some studies report applications related to surgical guide design and prosthetically driven implant positioning, reflecting their incorporation into digitally assisted planning processes [41]. Reported outcomes in this area are heterogeneous and depend on the specific planning task investigated. Some studies evaluate performance using classification or prediction metrics, while others describe feasibility or agreement with clinician-generated plans [42]. Due to this variability, the literature does not provide a single standardized metric for assessing performance in implant planning.

A further characteristic of the available evidence is that many studies are based on retrospective datasets, simulation environments, or proof-of-concept designs. In several cases, models are developed to address specific components of the planning process that can be standardized, rather than the full complexity of clinical decision-making [43].

### 3.3. AI in Intraoperative Guidance and Surgical Support

Within implant dentistry, the intraoperative use of AI remains less extensively investigated than diagnostic and planning applications, although it represents an emerging component of digitally assisted surgical workflows. The available literature primarily describes its role in association with computer-assisted surgery, including dynamic navigation systems and, to a more limited extent, robotic-assisted implant placement [44,45,46].

Several studies report the integration of outputs derived from preoperative analysis into navigation-based systems designed to support implant positioning during surgery [47]. In these workflows, information obtained from radiographic evaluation and digital planning is transferred to intraoperative platforms and combined with real-time tracking technologies [48]. These systems enable continuous visualization of surgical instruments in relation to patient anatomy and the planned implant trajectory, typically based on CBCT datasets and optical or electromagnetic tracking mechanisms. In this setting, AI is not described as an autonomous intraoperative decision-making system. Rather, its role is generally described within broader digital systems, where algorithm-based processes may be involved in tasks such as data processing, image-to-patient registration, and alignment between preoperative planning and intraoperative execution [10,11]. The literature characterizes these applications as components of integrated workflows aimed at facilitating the translation of planning data into surgical actions [49].

Robotic-assisted implantology represents a further area of investigation, although the available evidence remains limited [50]. A small number of studies describe systems in which robotic platforms are used to support implant placement, often relying on predefined trajectories derived from digital planning [51]. In such contexts, some studies describe the use of algorithm-based components in motion control, system calibration, or adaptation of predefined procedural parameters [52]. These applications are typically evaluated under controlled conditions. In addition to navigation and robotic systems, some reports describe the broader integration of AI within intraoperative digital environments, particularly in relation to real-time data processing and feedback. These may include the integration of imaging data, instrument tracking, and procedural information within unified interfaces, allowing continuous correspondence between planned and executed surgical steps [11,14].

Outcomes reported in intraoperative applications vary according to the type of system investigated. Navigation-based studies commonly assess positional accuracy, including deviations between planned and placed implants, as well as feasibility and reproducibility [53]. Robotic systems are typically evaluated in terms of technical precision and consistency under standardized conditions [54]. Comparisons across studies remain limited due to variability in system design, validation protocols, and outcome measures. A further characteristic of the literature is that many intraoperative applications are evaluated in experimental, simulation-based, or pilot clinical settings. In several cases, the focus is placed on technical performance rather than on long-term clinical outcomes. Most intraoperative applications were evaluated in controlled, experimental, or pilot settings, with a primary focus on technical performance.

### 3.4. AI in Outcome Prediction and Risk Assessment

The use of AI for outcome prediction and risk assessment has been increasingly explored in implant dentistry, particularly in relation to the analysis of patient-specific clinical and radiographic data. Within the available literature, these approaches are primarily based on predictive modeling techniques aimed at estimating the probability of specific outcomes [10].

A growing number of studies describe the development of machine learning models trained on heterogeneous datasets that may include demographic variables, medical history, radiographic findings, and implant-related parameters [55]. These models are designed to identify patterns within complex datasets and generate probabilistic estimates regarding outcomes such as implant survival, risk of failure, or occurrence of peri-implant complications [56]. In this context, predictive approaches are typically based on supervised learning methods, where models are trained using labeled datasets to establish relationships between input variables and clinical outcomes [55].

Most of the available literature focuses on the prediction of implant survival and failure. In these studies, models are developed to estimate the likelihood of successful osseointegration or to identify variables associated with implant loss [57]. The parameters included in predictive models vary across studies and may encompass systemic conditions, bone-related characteristics, implant design, and surgical factors. In addition to survival prediction, several investigations address the assessment of peri-implant conditions [58]. These applications include models aimed at identifying or estimating the risk of peri-implant bone loss and peri-implant disease based on radiographic and clinical data [29]. In such contexts, predictive modeling is often associated with pattern recognition within imaging datasets, as well as with the integration of clinical variables [10]. Some studies further describe risk stratification approaches, in which patients are categorized according to their probability of developing complications or experiencing unfavorable outcomes [59]. These models are based on combinations of patient-specific variables and are used to generate categorized risk profiles.

Model performance in outcome prediction studies is commonly evaluated using metrics such as accuracy, area under the receiver operating characteristic curve (AUC), sensitivity, and specificity [57]. Reported performance varies across studies, and differences in dataset characteristics, variable selection, outcome definitions, and validation strategies are described in the literature [60]. Most studies are based on retrospective datasets, frequently derived from single-center cohorts [59]. These datasets often include variations in sample size, follow-up duration, and completeness of clinical information. Differences in input variables and outcome measures are also reported across studies [61]. In several investigations, predictive models are based on complex architectures, and the contribution of individual variables to model outputs is not always explicitly defined [57,58]. These factors contribute to variability in the reported performance of predictive models across studies.

Table 1 summarizes the principal applications of artificial intelligence in implant dentistry, highlighting the most adopted AI approaches, evidence and validation characteristics, reported outcomes, major limitations, and the current stage of evidence regarding clinical applicability.

## 4. Discussion

### 4.1. Interpretation of Findings and Clinical Implications

The distribution of AI applications across implant dentistry does not appear uniform, with a clear concentration of studies in the diagnostic domain. This trend likely reflects the nature of the available data, as imaging datasets provide structured inputs that are particularly suited to pattern recognition techniques. Consequently, image-based analysis represents the area in which algorithmic models have achieved the highest level of technical development [45]. Despite this, the transition from technical performance to clinical utility remains less straightforward. High diagnostic accuracy reported under controlled conditions does not necessarily translate into consistent performance in daily practice, where variability in image acquisition, anatomical presentation, and clinical context may influence the reliability of AI-supported outputs [41]. Commonly reported metrics such as accuracy, sensitivity, specificity, Dice coefficient, and area under the curve primarily reflect technical performance and agreement with reference standards. However, high values for these parameters do not necessarily imply improved treatment planning, reduced surgical complications, enhanced clinical decision-making, or superior long-term implant outcomes. Therefore, technical performance metrics should not be considered direct indicators of clinical benefit. Demonstrating real-world applicability requires external validation across different patient populations, imaging protocols, and clinical environments, as well as evidence that AI-assisted systems can improve diagnostic reliability, treatment planning, or patient-centered outcomes beyond algorithmic performance alone. This distinction reflects a broader gap between technical performance and clinical utility. Most AI systems are evaluated using metrics such as accuracy, sensitivity, specificity, or AUC, which describe algorithmic behavior but do not necessarily demonstrate improvements in diagnostic reliability, treatment planning quality, or clinical outcomes. In addition, the predominance of retrospective and single-center datasets limits the generalizability of these findings. Models trained under controlled conditions may not maintain the same level of performance across different patient populations, imaging protocols, or clinical environments. In this regard, the current role of these systems seems more aligned with assisting interpretation rather than redefining it. When considering the planning phase, a different pattern emerges. Rather than addressing treatment planning, most approaches are directed toward specific components of the decision-making process [62]. This reflects the intrinsic complexity of implant planning, which requires the integration of prosthetic objectives, anatomical constraints, and patient-related factors [10,45]. Attempts to model such complexity through predictive systems remain limited, and current applications appear more effective when restricted to clearly defined and standardized tasks.

A further shift is observed in the implantology intraoperative setting, where the contribution of AI becomes less visible and more dependent on the surrounding technological framework [63]. Navigation systems and robotic platforms incorporate algorithm-based processes that facilitate the execution of preoperative planning, yet the decision-making process itself remains largely under clinician control [48]. In this phase, the emphasis is placed on precision and reproducibility, rather than on autonomous interpretation. Predictive modeling introduces an additional layer of complexity, as it moves from the analysis of existing data toward the estimation of future outcomes [10]. While these approaches offer a structured method for identifying potential risk patterns, their outputs remain inherently probabilistic and strongly influenced by the characteristics of the underlying datasets [14]. The variability in model design, input variables, and outcome definitions further complicates the interpretation of predictive performance across studies [39]. When considered together, these different domains highlight a progressive shift in the role of AI across the implant workflow. Applications tend to be more structured and technically robust in phases where data are standardized and measurable, such as imaging analysis, whereas they become more variable and context-dependent in phases requiring clinical interpretation and decision-making [64]. Accordingly, most of the available evidence can be interpreted as reflecting either experimental or technically validated applications, rather than fully clinically applicable systems. Only a limited number of studies approach real-world clinical validation. This gradient reflects not only differences in technological maturity but also the inherent complexity of translating algorithmic outputs into clinically actionable strategies.

From a broader clinical perspective, the integration of AI appears closely linked to the progressive digitalization of implant dentistry [65]. Rather than representing a standalone innovation, these systems are embedded within a network of technologies that includes advanced imaging, digital planning tools, and data integration platforms. Their contribution is therefore best understood as part of a wider transformation in how clinical information is processed and utilized [66]. At present, the available evidence does not support the view of AI as an autonomous clinical actor. Its role remains complementary, with the clinician retaining responsibility for interpretation, decision-making, and treatment execution. The added value of these systems lies in their ability to process large volumes of data and to support specific analytical tasks, particularly in areas where a certain degree of standardization can be achieved. This suggests that the current evolution of AI in implant dentistry is characterized less by the replacement of clinical expertise and more by a reconfiguration of how information is managed within the clinical workflow [67]. The extent to which this transformation will translate into measurable improvements in patient outcomes remains closely linked to the integration of these systems within real-world clinical settings.

### 4.2. Medico-Legal Implications of AI in Implant Dentistry

The progressive incorporation of AI into implant dentistry introduces a series of medico-legal considerations that extend across all phases of the clinical workflow, from diagnosis to outcome prediction [68]. These implications are closely related to the socio-technical nature of AI systems, which operate within human-defined contexts and are shaped by the data, assumptions and design choices embedded in their development [69]. For this reason, the use of AI in clinical practice cannot be regarded as a purely technical process, but rather as an interaction between algorithmic outputs and professional responsibility.

One of the central issues concerns the attribution of responsibility in the event of diagnostic or therapeutic errors. Regardless of their level of sophistication, AI-based systems do not possess legal or ethical agency [70]. Consequently, accountability remains with the clinician, who is required to interpret and validate algorithm-generated outputs within the clinical context.

This becomes particularly relevant across the different stages of implant treatment [70]. In diagnostic settings, discrepancies between AI-assisted image interpretation and clinician assessment may introduce uncertainty regarding the correct identification of anatomical structures or pathological conditions. During treatment planning, reliance on algorithm-supported recommendations may influence decisions regarding implant positioning or surgical approach [17,18]. In the intraoperative phase, where AI is embedded within navigation or robotic systems, errors in data alignment or execution may raise complex questions regarding the distribution of responsibility between clinician and technology.

From a regulatory perspective, the clinical use of AI in implant dentistry should be interpreted within existing frameworks governing medical devices and data protection. In the European context, AI-based systems intended for diagnostic or therapeutic purposes may fall within the scope of the Medical Device Regulation (EU 2017/745), requiring demonstration of safety and clinical performance [71]. At the same time, the processing of patient data must comply with the General Data Protection Regulation, particularly with regard to data protection, transparency, and patient rights. More recently, the European Artificial Intelligence Act has introduced a risk-based classification of AI systems, with healthcare applications often considered high-risk, requiring human oversight, traceability, and robustness [71]. The high risk is associated with the potential impact that AI has on clinical decisions. Despite this impact, clinicians retain full responsibility for each final therapeutic decision, which they must exercise critical judgment and maintain constant monitoring. Europe has therefore established a comprehensive regulatory framework for AI in healthcare based on three main pillars: the Medical Device Regulation (EU 2017/745), General Data Protection Regulation and the AI Act. However, regulatory approaches differ across international jurisdictions. In the United States, AI-based medical software is primarily regulated by the Food and Drug Administration (FDA), with particular emphasis on clinical validation, post-market surveillance and the management of algorithm updates over time. Unlike the more prescriptive European framework, the United States regulatory model places greater emphasis on adaptive oversight and lifecycle management of software performance. Other countries in Asia adopt heterogeneous regulatory strategies. However, most systems converge on core principles of patient safety, clinical validation, and the requirement for human oversight in clinical decision-making.

Data quality represents a critical factor frequently discussed in the literature, with direct medico-legal implications [19]. The performance of AI systems is strongly dependent on the characteristics of the datasets used for their development, including their completeness, representativeness, and accuracy. In implant dentistry, where patient-specific variables significantly influence treatment outcomes, the use of biased or non-representative data may lead to inaccurate estimations or inappropriate recommendations [10]. Such limitations may not always be immediately evident, particularly in complex models, making it more difficult to identify the origin of potential errors and to attribute responsibility in the event of adverse outcomes.

Closely related to this issue is the problem of algorithmic transparency. Many AI systems, especially those based on deep learning architectures, operate as “black-box” models, where the internal decision-making process is not easily interpretable [72]. This lack of explainability may limit the clinician’s ability to critically assess the validity of AI-generated outputs, with implications for both clinical decision-making and medico-legal accountability. In situations where clinical decisions are influenced by non-transparent systems, the justification of those decisions may become more challenging, particularly in legal contexts where traceability and reasoning are essential [73].

The use of AI in implant dentistry also raises important considerations regarding patient autonomy and informed consent [17]. The integration of algorithm-based tools into diagnosis, planning, or risk assessment introduces an additional layer of complexity in the communication process. Patients should be adequately informed about the role of AI in their care, including its potential benefits, limitations, and associated uncertainties. In clinical practice, this aspect may be particularly challenging, as patients are often unfamiliar with the underlying functioning of algorithm-based systems [74]. This is especially relevant in predictive applications, where the communication of probabilistic outcomes may influence patient perception and decision-making, potentially affecting acceptance of treatment. From a clinical perspective, the integration of AI-based tools into implant dentistry requires clear procedural and documentation standards. Clinicians should document the specific AI systems used, their clinical purpose and the extent to which AI-generated outputs contributed to diagnostic or treatment decisions. AI-assisted outputs should always be critically evaluated and cross-checked against clinical examination, radiographic findings and professional judgment. Furthermore, the verification process should be explicitly incorporated into clinical workflows to ensure traceability and accountability. In terms of informed consent, patients should be clearly informed about the use of AI technologies in their diagnostic or treatment planning process, including their role, limitations, and potential uncertainties. This practical framework reinforces transparency, supports medico-legal accountability and ensures that AI operates as a supportive tool within clinician-led decision-making rather than as an autonomous decision-maker.

In the specific context of implant dentistry, these regulatory requirements have practical implications for the use of AI-assisted systems. For example, AI tools applied to CBCT interpretation, anatomical segmentation, implant planning, or surgical navigation may be considered software as a medical device when they influence diagnostic or therapeutic decisions. In such cases, compliance with regulatory standards requires not only technical validation but also documentation of clinical performance, traceability of data sources, and a clear definition of the intended use.

From a data protection perspective, the use of CBCT scans, intraoral scans, and patient clinical records for AI development or deployment requires particular attention to data anonymization, storage, and processing conditions. This is especially relevant when cloud-based platforms or third-party software are involved, as data transfer and processing may occur across different jurisdictions.

Moreover, the classification of AI systems as high-risk technologies under the European regulatory framework implies that clinicians should be aware of the level of autonomy of the system, the presence of human oversight, and the transparency of the decision-making process. In implant dentistry, where treatment decisions involve multiple patient-specific variables, the lack of explainability may represent a critical limitation, particularly in medico-legal scenarios involving complications or adverse outcomes [71].

Data protection and privacy represent further areas of concern frequently highlighted in the literature [75]. AI systems rely on the processing of large volumes of clinical and imaging data, which often include sensitive patient information. The management, storage, and sharing of such data must comply with applicable regulatory frameworks, and appropriate safeguards are required to prevent unauthorized access or misuse. In addition, the ability of AI systems to infer information from apparently non-sensitive data raises questions regarding the boundaries of personal data and the potential impact on patient privacy [75]. Another relevant aspect involves the risk of bias and discrimination. If the data used to train AI systems do not adequately represent the diversity of the patient population, the resulting models may produce outputs that are less accurate for specific groups [76]. In implant dentistry, this may affect diagnostic interpretation, treatment planning, or risk estimation, potentially leading to unequal standards of care. From a medico-legal perspective, such disparities raise concerns related to equity, fairness, and professional responsibility.

The implementation of AI in clinical practice also requires continuous monitoring and validation. Unlike traditional medical devices, AI systems may evolve over time, particularly when data-driven models are used. This dynamic nature necessitates ongoing assessment of performance, safety, and reliability, as well as mechanisms for identifying and managing potential errors [77]. The absence of structured monitoring processes may increase the risk of undetected system failures, with potential legal consequences. Finally, the increasing integration of AI into implant workflows underscores the importance of professional competence and training. Clinicians are expected not only to use these technologies but also to understand their underlying principles, limitations, and potential sources of error [77]. This requirement reflects a shift in professional responsibility, where clinical expertise must be complemented by a critical understanding of digital tools and algorithmic systems.

These considerations reflect how medico-legal challenges arise from the interaction between technology, data, and clinical decision-making, rather than from any single component of the implant workflow.

### 4.3. Limitations of AI in Implant Dentistry and Future Perspectives

The interpretation of the current literature on AI in implant dentistry requires careful consideration of several methodological and translational limitations that are inherent to this field. A substantial proportion of the available studies is based on retrospective datasets, randomized and comparative trials or pilot studies conducted under controlled experimental conditions, which only partially reproduce the variability encountered in routine implant practice [59,78,79]. Clinical decision-making in implant dentistry is influenced by a complex interaction of anatomical, biological, and patient-specific factors. Elements such as variations in alveolar bone morphology, differences in bone density, and the proximity to critical anatomical structures introduce levels of variability that are difficult to fully capture within model development and validation processes [14]. This limitation becomes particularly relevant when considering the heterogeneity of study designs and outcome measures reported in the literature [39]. A central limitation of the current evidence is that many studies remain at the model-development stage and rely on retrospective datasets derived from single institutions, which may limit generalizability and lead to overestimation of performance. In addition, the absence of standardized reference standards represents a critical issue. In imaging studies, ground truth is frequently based on expert annotation, which may vary depending on operator experience and diagnostic criteria.

AI applications in implant dentistry encompass a wide range of tasks, including radiographic segmentation, implant site assessment, treatment planning, and outcome prediction [4]. Each of these applications is typically evaluated using different methodological frameworks, making it challenging to directly compare results across studies or to define consistent benchmarks for performance [42]. In the context of implant planning, for example, the determination of optimal implant positioning extends beyond anatomical assessment and requires the integration of prosthetic objectives, functional considerations, and clinician experience [80]. These aspects are only partially represented in current AI models. The issue of data representativeness further complicates the interpretation of reported findings. Many AI systems are developed using relatively homogeneous datasets, which may limit their applicability across diverse clinical settings and patient populations [8]. Therefore, the reported performance of AI systems in controlled datasets may not fully reflect their reliability in real-world clinical environments [81].

An additional aspect concerns the distinction between technical performance and clinical relevance [9]. Another important limitation concerns the limited evaluation of AI systems within real clinical workflows. Many studies assess algorithm performance in isolation, without considering their impact on clinician decision-making or patient outcomes. Future research should therefore prioritize external validation, multicenter datasets, and prospective clinical studies, focusing on clinically meaningful endpoints rather than technical performance alone.

While high levels of accuracy are often reported for tasks such as anatomical segmentation or implant identification, the extent to which these performances translate into improved clinical outcomes remains less clearly established. In implant dentistry, even minor deviations in implant positioning or inaccuracies in planning may have significant implications for prosthetic rehabilitation, biomechanical stability, and long-term peri-implant health [82]. Similarly, in the intraoperative phase, applications such as navigation-assisted implant placement depend on factors including registration accuracy and system calibration, which may influence the consistency between planned and executed procedures [12,13,53].

The integration of AI into implant workflows also raises practical and organizational considerations. Digital implantology already involves complex, multi-step workflows that include CBCT imaging, intraoral scanning, virtual planning, and, in some cases, guided or navigated surgery [45]. The introduction of AI-based tools within this framework has the potential to enhance specific components of the workflow but may also introduce additional layers of complexity [83]. Issues related to system interoperability, data management, and clinician interaction with technology play a crucial role in determining whether these tools effectively support clinical practice or contribute to workflow fragmentation [4]. Looking ahead, the future development of AI in implant dentistry will likely depend on the availability of larger, high-quality, and multi-center datasets capable of capturing the variability of clinical scenarios. The establishment of standardized protocols for imaging, data annotation, and model validation may contribute to improving comparability across studies and strengthening the overall evidence base. At the same time, increasing attention is being directed toward model interpretability, particularly in applications involving treatment planning and risk assessment, where understanding the rationale behind algorithmic outputs is essential for clinical adoption.

Another key area for future research concerns the design of prospective clinical studies that evaluate the real impact of AI on implant outcomes. Evidence regarding long-term results, complication rates, and patient-centered outcomes remains limited, and further investigation is required to determine whether AI-supported approaches provide measurable clinical benefits in everyday practice. In addition, the development of AI systems capable of integrating multiple sources of data, radiographic, clinical, and prosthetic, may represent an important step toward more comprehensive and clinically relevant decision-support tools. In this context, the evolution of AI in implant dentistry appears more closely linked to its progressive alignment with clinical complexity than to rapid technological replacement. Its future role will likely depend on the ability to integrate algorithmic capabilities within the nuanced and multifactorial nature of implant treatment, rather than on simplifying or standardizing it. Overall, current evidence suggests that AI in implant dentistry is progressing more rapidly at a technical level than at a clinical level, highlighting the need for stronger translational validation. Although the available findings are promising, many AI applications remain supported primarily by retrospective studies conducted on selected datasets and cannot yet be considered fully validated for routine clinical implementation. Additional prospective multicenter studies evaluating clinically meaningful outcomes are required to support evidence-based implementation of AI in implant dentistry. Finally, the rapidly evolving nature of AI research represents an inherent limitation of any review in this field. Regular updates of the literature will therefore be necessary to maintain an accurate and current understanding of the role of AI in implant dentistry.

## 5. Conclusions

Artificial intelligence is progressively being incorporated into implant dentistry, with applications extending across diagnosis, treatment planning, intraoperative support, and outcome prediction. At present, AI in implant dentistry should be regarded primarily as a decision-support technology rather than an autonomous decision-making system. The available evidence indicates a higher level of development in imaging-based tasks, where structured data enable reliable pattern recognition, whereas applications involving complex clinical decision-making remain more variable and context-dependent. Within the implant workflow, AI primarily acts as a supportive tool, contributing to the analysis and processing of radiographic and clinical data. Its role in treatment planning and outcome estimation remains influenced by data quality, methodological variability and the need for continuous clinician oversight, particularly in individualized clinical scenarios.

The increasing integration of AI into digital implant workflows may improve procedural consistency and data management. However, the heterogeneity of existing studies, the lack of standardized validation frameworks, and the limited availability of prospective clinical evidence highlight the importance of cautious interpretation and implementation. The use of AI in implant dentistry also raises relevant medico-legal considerations, including issues related to professional responsibility, data governance, algorithmic transparency, and patient autonomy, which remain central to its safe clinical adoption.

Future developments will depend on the availability of robust and representative datasets, the standardization of methodological approaches, and the generation of clinically oriented evidence. In this context, AI is unlikely to replace clinical expertise, but rather to reshape the way information is integrated within the implant workflow, reinforcing the central role of the clinician in complex decision-making processes.

## Figures and Tables

**Table 1:** Overview of the current evidence on artificial intelligence applications in implant dentistry, including AI approaches, evidence and validation characteristics, reported outcomes, major limitations, and current stage of evidence.

Application Area	Common AI Approaches	Typical Validation Approach	Main Clinical Applications	Reported Outcomes	Major Limitations	Current Stage of Evidence
**Diagnostic imaging and radiographic analysis**	Convolutional Neural Networks (CNNs), Deep Learning (DL), image segmentation algorithms	Predominantly retrospective studies with internal validation and comparison against expert reference standards	Detection and segmentation of anatomical structures, mandibular canal identification, maxillary sinus analysis, implant recognition, peri-implant bone assessment	High diagnostic accuracy and agreement with expert annotations in imaging-based tasks	Predominantly retrospective datasets, variability in imaging protocols, limited external validation, heterogeneous reference standards	Technically validated with emerging clinical applicability
**Implant planning and decision-support systems**	Machine Learning (ML), Deep Learning (DL), decision-support algorithms	Retrospective and simulation-based investigations, often focused on proof-of-concept validation	Bone assessment, implant site evaluation, implant positioning support, surgical guide planning, workflow automation	Support for specific planning-related tasks and improved integration within digital workflows	Limited ability to reproduce complex clinical reasoning, predominantly retrospective or simulation-based evidence	Technically validated
**Intraoperative guidance and surgical support**	AI-assisted navigation systems, robotic-assisted platforms, real-time tracking algorithms	Experimental studies, pilot clinical investigations, and controlled validation settings	Dynamic navigation, guided implant placement, robotic-assisted implant surgery	Improved positional accuracy and procedural reproducibility under controlled conditions	Limited prospective clinical validation, heterogeneous validation protocols, scarce long-term outcome data	Early clinical implementation with limited validation
**Predictive modeling and risk assessment**	Supervised ML models, predictive analytics, classification algorithms	Retrospective cohort-based studies with variable validation strategies and occasional multicenter datasets	Prediction of implant survival, peri-implant disease risk estimation, complication prediction, patient stratification	Promising predictive performance based on accuracy, sensitivity, specificity, and AUC metrics	Dependence on dataset quality, limited generalizability, lack of prospective and multicenter validation	Experimental to technically validated
**Medico-legal and ethical implications**	Explainable AI approaches, AI-assisted decision-support systems, governance frameworks	Conceptual, regulatory, and framework-based analyses rather than empirical validation studies	Professional accountability, informed consent, algorithm transparency, data governance, regulatory compliance	Increased awareness of legal, ethical, and regulatory challenges associated with AI implementation	Regulatory heterogeneity, black-box effect, unresolved liability attribution, limited practical guidance	Conceptual and regulatory framework

## Data Availability

No new data were created or analyzed in this study.
